# Comparison of Medical Adhesive Tapes in Patients at Risk of Facial Skin Trauma under Anesthesia

**DOI:** 10.1155/2016/4878246

**Published:** 2016-06-12

**Authors:** Ling Antonia Zeng, Sui An Lie, Shin Yuet Chong

**Affiliations:** Department of Anaesthesia, Singapore General Hospital, Block 2, Level 2, College Road, Singapore 169608

## Abstract

*Introduction*. Adhesive tapes are used for taping eyelids closed and securing endotracheal tubes during general anesthesia. These tapes can cause facial skin injury. We compared the incidence of facial skin injury and patient satisfaction with different tapes used.* Methods*. A total of 60 adult patients at risk of skin trauma were randomized to use 3M*™* Kind Removal Silicone Tape or standard acrylate tapes: 3M Durapore (endotracheal tube) and Medipore (eyelids). Patients were blinded to tape used. Postoperatively, a blinded recovery nurse assessed erythema, edema, and denudation of skin. Anesthesiologist in charge also assessed skin injury. On postoperative day 1, patients rated satisfaction with the condition of their skin over the eyelids and face on a 5-point Likert scale.* Results*. More patients had denudation of skin with standard tapes, 4 (13.3%) versus 0 with silicone tape (*p* = 0.026) and in anesthesiologist-evaluated skin injury 11 (37%) with standard versus 1 (3%) with silicone (*p* = 0.002). No significant differences were found in erythema and edema. Patient satisfaction score was higher with silicone tape: over eyelids: mean 3.83 (standard) versus 4.53 (silicone), Mann-Whitney *U* test, *p* < 0.001; over face: mean 3.87 (standard) versus 4.57 (silicone) (*p* < 0.001). *Conclusion*. Silicone tape use had less skin injury and greater patient satisfaction than standard acrylate tapes.

## 1. Introduction

Adhesive tapes are often used on the patient's face during general anesthesia. Taping the eyelids closed prevents corneal abrasions [1]. Adhesive tapes are also used to fix endotracheal tubes, temperature probes, gastric tubes, and nerve stimulator electrodes.

An adhesive tape used in anesthesia needs to provide fast, secure adhesion to prevent dislodgement of critical devices [2, 3]. The tape should be secure over time, with changes over temperature, humidity, or exposure to fluids [4] as occurring in the operating room. However, the tape should be gentle enough that removal should not cause skin trauma to the face and eyelids. Medical adhesive related skin injuries are estimated to impact at least 1.5 million patients annually in the US with significant costs per incident [5].

The skin over the face and especially eyelids is particularly susceptible as it is thinner, with poor barrier function. The stratum corneum to the eyelid also has poor skin surface lipids [6]. Facial skin injury including the eyelids is seldom studied but known complication of adhesive tapes used during general anesthesia [7–13]. Adhesive tapes have been examined on healthy volunteers but seldom in clinical use [14, 15] with the exception of premature infants.

Medical adhesive tapes are composed of several layers including a tape backing and an adhesive layer. Tape backing is often made of silk, cloth, or paper depending on bonding with adhesive and breathability of material. Common types of adhesives include acrylates, latex, hydrocolloids, polyurethane, and the newer silicone based adhesives. Medical adhesives are pressure sensitive, where firm pressure applied to the surface of the adhesive will increase surface area contact and thus activate the adhesive. Traditional adhesives will warm and flow to fill in gaps between adhesive and irregularities in the skin surface thus increasing the adhesive strength over time. Most adhesive tapes used in anesthetic practice are acrylate based adhesive tapes. Newer silicone based adhesives being softer have a lower surface tension to fill in gaps quickly to maintain a constant level of adherence [16, 17]. Silicone tapes have been studied on the forearms of healthy volunteers [18–21] and healthy children [22] and on venous ulcers [23] and shown to be gentler on skin in comparison to traditional adhesives including acrylic adhesive.

Our study aims to prospectively compare the 3M Kind Removal Silicone Tape (3M Company, St. Paul, MN, USA) versus standard acrylate based adhesive tapes (3M Durapore and Micropore tape) for facial skin injury and patient satisfaction in at-risk patients under general anesthesia.

Patients considered at risk for facial skin injuries in adults include the elderly [8, 24, 25], those with increased skin fragility such as patients on chronic steroid treatment or those having undergone skin resurfacing procedures or using prescription exfoliating agents [9, 16], also in the prone position [7, 26] or prolonged tape duration [27].

Our hypothesis is that the silicone tape may have better patient satisfaction and less skin trauma in these at-risk patients.

## 2. Materials and Methods

This prospective randomized controlled study was approved by the Singhealth Institutional Review Board and written informed consent was obtained from all patients before inclusion in the study.

### 2.1. Study Population

Adult patients planned for surgery under general anesthesia with the use of an endotracheal tube were screened for risk factors for skin injury over a 6-month period.

Inclusion criteria were patients who fulfilled any of the following: being above the age of 70 years, chronic steroid therapy, history of cosmetic resurfacing procedures or prescription exfoliating agents, expected duration of surgery of more than 4 hours, or prone position.

Excluded from the study were patients who were pregnant or with impaired cognition unable to give their own consent and those undergoing head and neck surgery.

### 2.2. Randomization and Blinding

**Figure 1:**
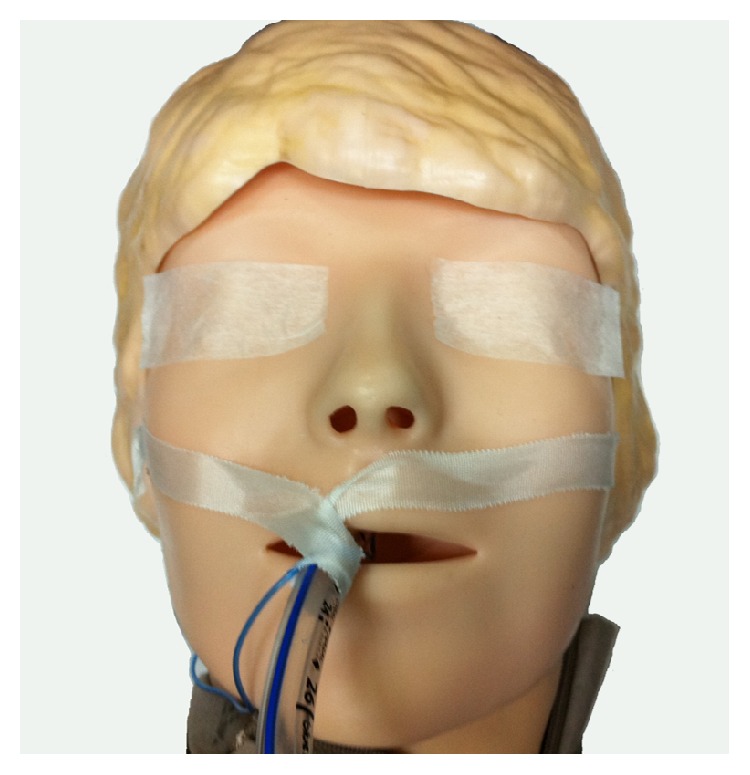
Use of tapes on the face.

Adult patients at risk of skin trauma were randomized to use either of the following:

1. standard acrylate tapes (3M Durapore for endotracheal tube and Medipore for eyelids),
2. silicone tape (3M Kind Removal) for eyelids and endotracheal tube,

for surgery under general anesthesia.  Figure 1 shows how the tapes are used on the face under anesthesia.

Randomization was done via computer generated random numbers program in a 1 : 1 ratio. Patients were kept blinded to tape used and the tapes were removed in the operating room.

### 2.3. Postoperative Assessment

Postoperative assessment of the skin is done by 2 assessors—the anesthesiologist in charge of the care of the patient, who is not blinded, as well as an independent assessor at the recovery unit. Patients were also followed up regarding their satisfaction with the condition of their skin.

The anesthesiologist in charge assessed the presence of any facial skin injury and filled up an evaluation form together with relevant data at the end of surgery. Tapes were removed prior to the transfer of the patient to the recovery unit.

In order to assess patients in the recovery unit, assistance from 2 recovery nurse clinicians was obtained for the study. They evaluated the patient's skin for erythema, edema by a score of 0–4, and skin denudation by a score of 0–4 as described in the following part (adopted from other studies for skin injury [18, 22, 28]). For standardization, both nurse clinicians were briefed about assessment for skin injuries and given pictorial examples of the different severities of injury prior to start of the study. The assessors at recovery were kept blinded to type of tape used.

*Severity Scoring for Skin Injury*

*Erythema and Edema Severity Score*. Consider the following:

0: no visible response.
1: mild response; diffused, patchy, not well defined, and just barely perceived erythema; no perceivable edema.
2: moderate response, perceivable erythema obvious, with diffused redness; pink or red color, area well defined; no edema.
3: severe response; obvious erythema; definite red color, area well defined; edema present.
4: extreme response; bright, fiery red erythema; edema present.

*Denudation Severity Score*. Consider the following:

0: no sign of denudation.
1: trace amount of denudation in epidermis (slight glazed appearance).
2: partial thickness denudation (first sign of pitting in the skin) extending up to the glistening layer of the epidermis (moist and/or wet surface).
3: full thickness denudation extending into the dermis (exudates present).
4: full thickness denudation extending into the dermis or in combination with an extreme.

Patients were followed up on the 1st postoperative day by one of the investigation team members. Patients rated satisfaction with the condition of their skin over the eyelids and face on a 5-point Likert scale.

### 2.4. Sample Size

Due to the lack of prior experience with measuring the effects of the adhesive tape for use in anesthesia, power calculations were based on previous studies on endotracheal tube securement [29–31].

Satisfaction scores on a 5-point Likert scale in these studies had a standard deviation of 0.8–1.0. For type 1 error rate of 0.05 and power of 0.8, to detect a difference in score of 1 would require 16 patients per group. To allow for error in estimation of the standard deviation, nonparametric analysis, and dropouts, a target sample size of 30 patients in each group was set.

Studies comparing the silicone tape to other adhesives in healthy volunteers also used less than 30 patients per group [18, 22].

### 2.5. Statistical Analysis

The erythema and edema severity score and denudation severity score were planned to be analyzed using nonparametric tests. However due to the very low numbers of moderate and severe skin injuries, the data was grouped instead into the presence of erythema and edema and for the presence of denudation and analyzed using contingency tables with Fisher's exact test. The presence of skin injury as evaluated by the anesthesiologist was compared using Fisher's exact test. Patient satisfaction scores were compared with Mann-Whitney *U* test.

All statistical analyses were performed using SPSS version 20 for Windows (SPSS Inc., Chicago, IL).

## 3. Results

**Figure 2:**
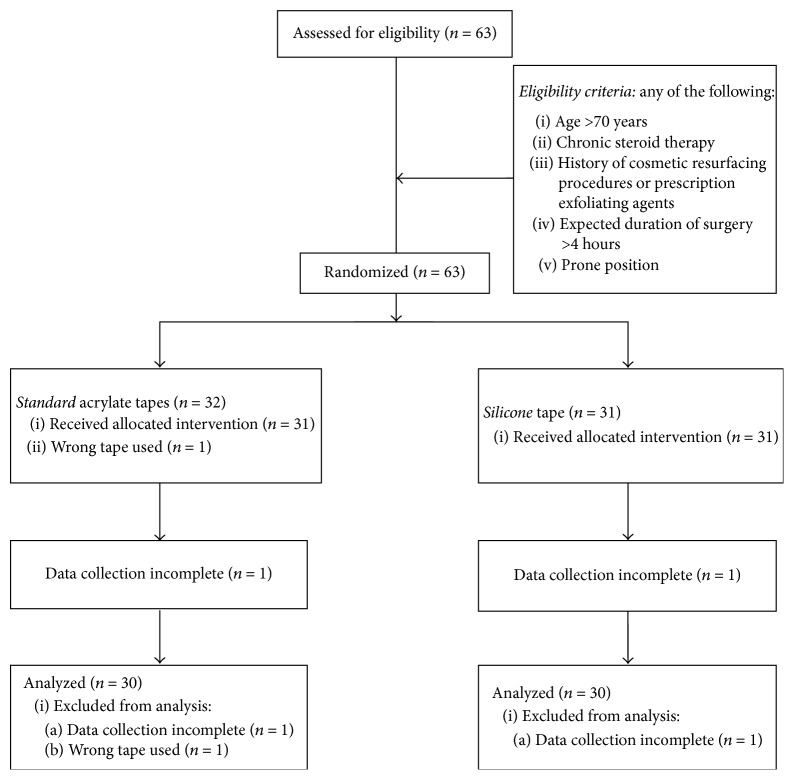
CONSORT flow diagram.

A total of 63 patients were recruited for the study: 2 were excluded from analysis due to incomplete data and 1 due to an error in protocol—wrong tape was used. See Figure 2 for CONSORT flow diagram; 30 patients were included in each group for the silicone tape and the standard acrylate tape. Patient characteristics are shown in Table 1.

**Figure 3:**
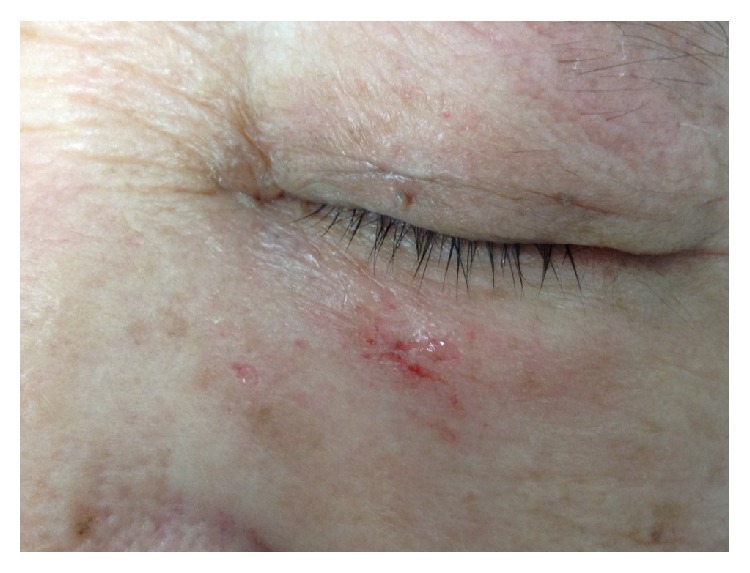
Example of skin denudation.

The results of severity scores for skin injury are shown in Table 2.  Table 3 describes the analysis of results. More patients had denudation of skin with standard acrylate tape: 4 of 30 (13.3%) versus 0 of 30 with silicone tape (*p* = 0.026).  Figure 3 shows an example of skin denudation experienced by one of the patients in the study. There was however no significant difference in erythema and edema of the skin.

For skin injury evaluated by the anesthesiologist, more injuries were seen with the standard acrylate tape: 11 patients (37%) with standard tape versus 1 patient (3%) with silicone (*p* = 0.001).

Patient satisfaction score was higher in the silicone tape group. There were no incidents of endotracheal tube dislodgement during the study.

## 4. Discussion

We found a decreased incidence of skin injury with the silicone tape versus the standard acrylate tapes. This was confirmed by both the anesthesiologist and an independent blinded assessor in the recovery unit. Patients also reported a higher satisfaction score in the silicone tape group compared to standard tapes and this was due to the presence of skin injuries in the standard tape group.

Although our study size was small, we selected patients at risk for skin injury and found a significant difference between the tapes used. Most of the patients included in the study were undergoing prolonged surgery in the prone position or were elderly. The prone position has also been associated with soft tissue injuries due to direct pressure [26]. The adhesive strength of acrylate tape also increases with time [27]; thus prolonged surgery would increase the risk of skin injuries during removal of the tape. With ageing, epidermal thinning occurs with loss of dermal matrix and subcutaneous tissue, reduced cohesion between the dermal and epidermal layers, and decreased elasticity, vascularity, and moisture [25, 32]. Many of the reports of more severe skin injuries with adhesive tapes in literature are also in the elderly [8, 13, 24].

Some factors we did not account for in the study that may impact skin injury include peel force during removal of tape, application pressure, and peel angle [33–35]. Our nurses and doctors are trained to apply and remove tape in a standardized fashion, but we were unable to have the same person perform removal of tapes for all patients in the study due to practical limitations and hence application and peel force were not standardized. It is difficult to comment whether the tapes would be applied or removed using the same force in patients when they are anaesthetized, as compared to when they are awake. However, peel force has not been consistently shown to account for skin damage, as low levels of peel force are not always associated with low damage [21].

The silicone tape however may be less moisture resistant than standard acrylate tapes [16]; thus in situations where the tape may be exposed to cleaning solutions or excessive secretions it may be prudent to use acrylate based adhesive tapes instead.

The morphological spectrum of medical adhesive related skin injuries is broad and can be classified into the following: (a) mechanical (skin/epidermal stripping, tension injury or blister, and skin tears); (b) dermatitis (irritant contact dermatitis, allergic dermatitis); and (c) others (maceration, folliculitis). The pathophysiology of medical adhesive related skin injuries is not fully understood. Skin injury occurs when the skin to tape adhesion is stronger than adhesive forces between the skin cells and skin layers so that they separate when the tape is peeled off [16]. Irritant contact dermatitis occurs when there is a biological response to certain components in the tapes, resulting in skin inflammation without production of specific antibodies. It is often classified according to morphology and clinical course [36]. Skin injury is not a rare anesthesia-related injury. In long term care facilities, it is reported as 15.5% incidence rate in the elderly [24]. It is quoted in standard anesthesia textbooks [11, 26] but there are few reports in the literature in anesthesia and even fewer studies on the subject. This may represent a publication bias as skin injuries may be seen as a recognized hazard of anesthesia.

With a growing elderly population and with rapidly advancing surgical procedures growing in complexity and duration, a suitable medical tape that both securely affixes devices to sensitive skin and minimizes skin trauma on removal is necessary. Since a roll of tape can be used for many patients, the cost of a 15 cm strip of the silicone tape from the 3M Shop [37] for one patient is US$0.12, while the Durapore tape is $0.02, which only makes a price difference of $0.10 per patient. In our study, 4 out of 30 patients developed skin denudation with the standard acrylate tapes while 0 out of 30 did with the silicone tape. It would seem worthwhile in the at-risk patient to invest that extra dime to avoid painful, distressing, and sometimes costly skin injuries.

## 5. Conclusion

We found significantly less skin injury and greater patient satisfaction with the 3M silicone tape versus standard acrylate tapes used on the face. The silicone tape is a promising alternative to current standard adhesive tapes used. It should be further evaluated in a general population not at high risk of skin injury.

## Figures and Tables

**Table 1:** Patient and surgery characteristics.

Characteristic	Standard acrylate tape (*n* = 30)	Silicone tape (*n* = 30)
Age (yrs) (median, range)	63.5 (21–83)	62.0 (27–75)
Cosmetic procedures	2	0
Chronic steroid therapy	0	1
Duration of surgery (hrs)	3.2 (1.6)	3.5 (1.8)
Prone position	26	25
Type of surgery		
Spine surgery	24	25
Plastic surgery	2	3
Others	4	2

Data are mean (standard deviation) or number unless otherwise stated.

**Table 2:** Severity of skin injury.

		Standard acrylate	Silicone	Total
Erythema and edema severity	None	15	20	35
	Mild	12	9	21
	Moderate	2	1	3
	Severe	1	0	1

	Extreme	0	0	0

Total		30	30	60

Denudation severity	None	26	30	55
	Mild	3	0	4
	Moderate	1	0	1
	Severe	0	0	0

	Extreme	0	0	0

Total		30	30	60

**Table 3:** Skin injury and patient satisfaction in different groups.

	Standard acrylate tape	Silicone tape	*p* value
Assessment in recovery for presence of			
(i) Erythema and edema	15 (50%)	10 (33%)	0.147
(ii) Denudation	4 (1.3%)	0 (0%)	**0.026**

Anaesthesiologist evaluated skin injury	11	1	**0.001**

Patient satisfaction score for skin over			
Eyelids	3.83 (0.69)	4.53 (0.51)	**<0.001**
Face	3.87 (0.70)	4.57 (0.50)	**<0.001**

Data are in number (percentage) or mean (standard deviation).
